# Abnormal Developement of the Liver and Spleen at Birth

**Published:** 1851-03

**Authors:** D. Hutchinson

**Affiliations:** Mooresville, Ind.


### ARTICLE III.

*Case of Abdomanal developement of the Liver and Spleen at birth*, By D. Hutchinson, M. D., of Mooresville, Ind.

On the morning of the 4th of June, 1850, I was called to visit Mrs. B. in labor, with her second child. She was a healthy young female, æt. 21 years. Her labor proceeded pleasantly, and in an hour after my arrival, she was delivered of a female child. The child appeared below the ordinary size for a mature fœtus, with the exception of the abdomen, which was excessively disproportionate to the head and extremities. The lady affirmed that she had gone the full period of utera-gestation. The heart and umbilical cord was still pulsating, it opened its eyes, yet it showed no symptoms of the establishment of respiration. It was only after considerable effort, by artificial respiration, kept up through a gum elastic catheter, introduced into the mouth of the child, that any appearance of breathing could be noticed. After partial respiration was established it attempted to cry, there was a considerable interval between each respiration. On examining the abdomen I found a firm and hard substance occupying the position of an enlarged liver, extending from the right hypochondriac regions to the middle of the epigastrium, and running with a well defined margin, down the median line of the abdomen, till it extended to the hypogastric and the illiac regions, occupying the entire right half of the abdomen. On the left side I found another hard substance, which appeared to correspond with the location and shape of an enlarged spleen, it extended over and met the liver at the median line of the abdomen, and descended into the hypogastric and left illiac regions, occupying the entire left half of the abdomen The abdomen appeared nearly double the size of a nine months fœtus, and the head and extremities were apparently imperfectly developed, or below the usual size. The breathing of the child continued slow and difficult, the lips and face shortly became of a purple color; black inky spots, about the size of pin heads, soon appeared over the body, the whole surface became purple, and in three quarters of an hour it died. I was not permitted to make any post mortem examination.

The abdominal regions appeared to have been developed at the expense of the head and extremities, and may probably be accounted for, by an abnormal formation of the circulating apparatus.

